# Effects of *MTHFR* gene polymorphism on the clinical and electrophysiological characteristics of migraine

**DOI:** 10.1186/1471-2377-13-103

**Published:** 2013-08-05

**Authors:** Julia E Azimova, Alexey V Sergeev, Liubov A Korobeynikova, Natalia S Kondratieva, Zarema G Kokaeva, Gadji O Shaikhaev, Kirill V Skorobogatykh, Natalia M Fokina, Gyusal R Tabeeva, Eugene A Klimov

**Affiliations:** 1 Department of Genetics, Faculty of Biology, Lomonosov Moscow State University, Moscow, Russia; 2 Department of Neurology and Clinical Neurophysiology, Scientific-Research Centre, Sechenov First Moscow State Medical University, Moscow, Russia; 3 University headache clinic, Moscow, Russia; 4 Vavilov Institute of General Genetics of Russian Academy of Sciences, Moscow, Russia; 5 Isogene Lab. ltd, Moscow, Russia

## Abstract

**Background:**

It was previously shown that the *MTHFR* gene polymorphism correlated with an increased risk of migraine, particularly migraine with aura. The substitution of cytosine for thymine at the position 677 of the *MTHFR* gene leads to formation of the thermolabile form of the protein and development of hyperhomocysteinemia, which increases the probability of migraine. The purpose of this study was to determine whether the replacement of C677T in the gene *MTHFR* influenced any particular symptoms of the disease.

**Methods:**

We have analyzed clinical and electrophysiological characteristics of 83 patients with migraine (migraine with aura (MA), 19 patients, and migraine without aura (MO), 64 patients, according to the ICHD-II (2003)) taking into account their genotypes of C677T variant of *MTHFR*.

**Results:**

We have shown that MA was significantly more prevalent among the T-allele carriers (37.2%), as compared to the СС genotype patients (0%), р < 0.0001. Patients with TT genotype were not only more likely to have accompanying symptoms (significant differences were found only for photophobia), but also more sensitive to migraine attack triggers. In RP-VEP test results we observed a trend that the T-allele carriers were presented with the decreased N75/P100 amplitudes and a positive habituation index, as compared to the СС genotype patients.

**Conclusions:**

Thus, according to our data, the *MTHFR* genotypes are associated with several clinical and electrophysiological characteristics of migraine.

## Background

Among all the genes associated with common migraine and migraine with aura, the *MTHFR* gene encoding the enzyme 5,10-methylenetetrahydrofolate reductase (MTHFR), which is located on chromosome 1 (1р36.3), is the most thoroughly studied one. The mutation in this gene is essentially a replacement of cytosine (С) for thymine (Т) in position 677 (rs1801133). At the time of manuscript preparation, two meta-analyses had been published [1,2] that provided convincing evidence demonstrating that the ТТ genotype of the *MTHFR* gene (*MTHFR* 677TT genotype) was associated with an increased risk for migraine with aura in Caucasian population. Besides, a meta-analysis conducted by Schurks et al. has shown that the *MTHFR* 677TT genotype was associated with an increased risk for both variants of migraine in non-Caucasian populations [3]. A study conducted in Russia, which included 72 patients with migraine (all Caucasians) living in the city of Moscow (9 with migraine with aura (MA), 63 with migraine without aura (MO), and 50 healthy volunteers), has demonstrated that the occurrences of TT homozygosis and the T-allele of *MTHFR*677 were significantly higher in migraine patients (19.5% and 38.9%, respectively), as compared to controls (8.5% and 28%, respectively) [4]. Therefore, in the Russian population, *MTHFR* C677T polymorphism may be a genetic risk factor for the occurrence of both migraine with aura and migraine without aura. Similar results were obtained by Kowa et al. who demonstrated that the incidence of the homozygous transition (T/T) in the migraine patients (20.3%) was significantly higher than that in controls (9.6%), and the occurrence of the T/T genotype in the individuals with migraine headache with aura was remarkably high (40.9%) [5]. In a Turkish population, the occurrence rates of the T allele of *MTHFR* C677T were significantly higher in the total migraine population (33.82%) than those in controls (25.38%) [6]. These data are comparable with the results of the Russian study.

The aim of this study was to evaluate the effects of the *MTHFR* genotypes on the clinical symptoms of migraine and on the electrophysiological characteristics of migraine patients.

## Methods

### Patients

Eighty-three Caucasian consecutive unrelated out-patients living in the city of Moscow counseled at the Clinic of Nervous Diseases of the 1^st^ Moscow Medical University were enrolled in the study. The inclusion criteria were:

migraine (migraine with aura or migraine without aura (MO), according to the ICHD-II (2003));

the age of 18–69 years.

The exclusion criteria were:

probable migraine, according to the ICHD-II (2003);

familial or sporadic hemiplegic migraine, according to the ICHD-II (2003);

other significant medical conditions except migraine.

The control group was composed of 50 unrelated healthy Caucasians (healthy volunteers), living in the city of Moscow (without diagnosis of migraine or other type of headache). The two groups were comparable with regard to the gender and age distribution.

All the patients underwent a neurological interview and examination. Clinical information with regard to migraine characteristics was extracted from our database. The refractory migraine criteria proposed by Schulman et al. [7] were used.

Blood samples were collected by a qualified phlebologist under full ethical clearance by the Local Ethics Committee of Vavilov Institute of General Genetics RAS for experimentation on human subjects. Written informed consent was obtained from all the participants.

### Electrophysiology

From the entire group of patients, a subgroup without any preventive therapy in the previous 3 months was specified for a neurophysiological analysis (n = 22) based on RR-VEP in the inter-attack period (72 hours prior to or after migraine attack). All the patients who had participated in the analysis demonstrated no vision pathology.

The RP-VEP test was carried out in a period free from migraine attacks; it included recording of the five consecutive series of reversal-pattern visual evoked potentials; every series consisted of 100 mean values. RP-VEP were registered using leads O1 and O2 and reference electrode Cz. The RP-VEP test protocol was compiled according to the recommendations of the EUROHEAD scientific group (EUROHEAD Project) on conducting neurophysiological studies in patients with migraine. Stimulation was attained using an alternating (right – left eye) monocular black-and-white reversal pattern; distance to the monitor: 119 cm, check size: 28’ checks, stimulation frequency: 3.1 Hz, analysis time: 500 msec, number of means in a series: 100. RP-VEP were recorded with a Neuron-Spektr 4 VPM system (Neurosoft, Russia).

The N75-P100 response amplitude was evaluated in the 5 consecutive series of stimulation, mean values were obtained for the right eye and the left eye responses, the total N75-P100 amplitude was calculated for all 500 means, as was the habituation index (N75-P100 amplitude per cent change for series 5 versus the N75-P100 amplitude for series 1).

### Genetic examination

Isolation of genomic DNA from whole blood samples was performed using the Magna™ DNA Prep 200 kit (Isogene Lab. ltd, Russia). Allele-specific PCR was carried out using a GenePak® MTHFR PCR test kit (Isogene Lab. ltd, Russia), which is designed to determine the C677T point mutation in the *MTHFR* gene. The PCR conditions included initial denaturation (95°C for 1 min), followed by 15 cycles of denaturation (95°C for 20 sec), primer annealing (65°C for 20 sec), chain elongation (74°C for 40 sec); and 35 cycles with the same sequence, but with a primers annealing temperature of 55°C for 10 sec, the reaction was completed with the final elongation (72°C for 10 min). PCR products were separated in a 2% agarose gel.

### Statistical analyses

Statistical analyses were chosen depending on the sample distribution and included the parametric Student’s and Fisher’s tests or the non-parametric Wilcoxon and Kolmogorov – Smirnov tests. N75-P100 amplitude values were compared between the groups using Student’s t-test and Wilcoxon w-test. Single-factor ANOVA was employed to assess N75-P100 amplitude changes and to evaluate habituation. Pearson’s test (for the normal sample) and Spearman’s test (rank correlation coefficient) were used for the correlation analysis. Results were considered significant at p < 0.05.

## Results

19 patients had migraine with aura (MA), 64 patients – migraine without aura (MO). 73 of them were females. All the patients had moderate or severe migraine (MIDAS Grade III/IV).

Resulting allele and genotype frequencies for C677T locus in the samples of patients with diagnosed migraine and the control samples are presented in Table 1. Observed distribution of genotype frequencies in the studied samples for C677T locus corresponds to theoretically expected Hardy-Weinberg equilibrium (χ2=0.003; p=0.95 for control and χ2=2.74; p=0.10 for patients). Frequency of T allele in the migraine patients is significantly higher than in healthy individuals (χ2=4.96; d.f.=1; p=0.026; OR=1.829; 95% C.I .=[1.072-3.122]). These results are consistent with the data obtained earlier in other associative studies of C677T substitution [6,8,9].

**Table 1:** **The frequencies of occurrence of single nucleotide polymorphism C677T in *MTHFR *gene in sample of migraine patients and control sample**

**C677T**	**Genotype frequencies (P)**			**Allele frequencies (P±S(p))**	
	**CC**	**CT**	**TT**	**C**	**T**
Migraine patients (n=83)	0,385	0,398	0,217	0,584±0,054	0,416±0,054
Control sample (n=50)	0,520	0,400	0,080	0,720±0,072	0,360±0,072

The СС genotype occurrence in the migraine patients was found to be 38.5% (32 individuals), that of the СТ genotype was 39.8% (33 patients), and that of the ТТ genotype was 21.7% (18 individuals). Among healthy individuals, the СС genotype was found in 52.4% of the patients, the СТ genotype was observed in 39.1% of the study subjects, and the ТТ genotype was obtained in 8.5% of all the patients. Migraine patients with different genotypes did not differ in gender or age distribution. MA was significantly more frequent in T-allele carriers (37.2%), as compared to СС genotype patients (0%), р < 0.0001 (Table 2).

**Table 2:** **Clinical and demographic characteristics of patients with different *MTHFR *genotypes**

**Parameter**	**СС genotype**	**СТ genotype**	**ТТ genotype**	**Т-allele carriers**
Number of patients, %	32 patients, 38.5%	33 patients, 39.8%	18 patients, 21.7%	51 patients, 61.4%
Age (years)	41.9 ± 11.8	42.8 ± 13.7	47.9 ± 12.8	43.4 ± 13.6
Migraine with aura / migraine without aura, %,	0 patients / 32 patients 0/100	14 patients / 19 patients 42.4/57.6	5 patients / 13 patients 27.7/72.3	19 patients / 32 patients 37.2/62.8

All the patients with refractory migraine had migraine without aura. In the patients with CC genotype, Refractory migraine was observed in 12% of CC, 24% of CT, and 36.4% of TT genotype patients. The prevalence of refractory migraine among patients with TT genotype was significantly higher (p<0.01), as compared to CC and CT genotype patients.

**Figure 1:**
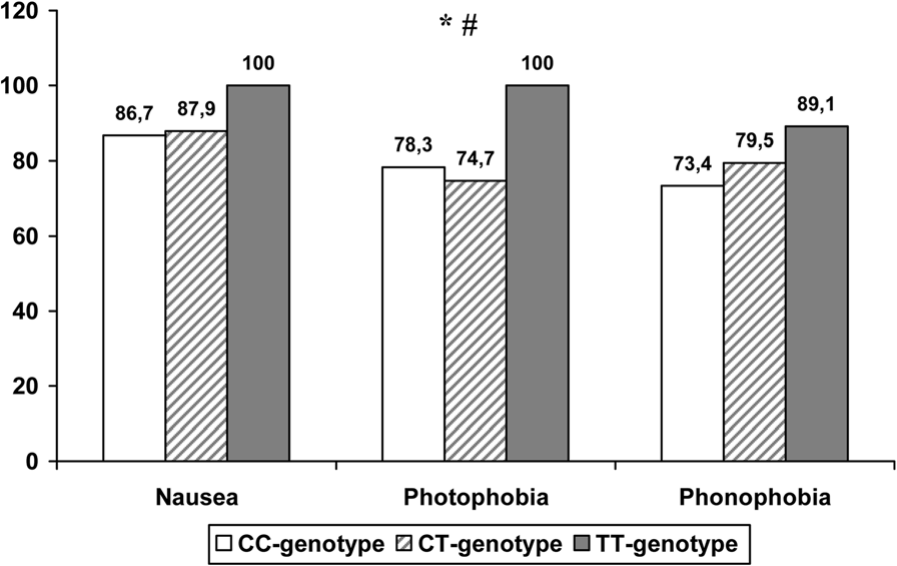
**Occurrence of migraine accompanying symptoms in patients with different genotypes of *MTHFR*.** * - p<0.05 CC vs TT, # - p<0.05 CT vs TT.

Notwithstanding the fact that patients with different genotypes did not significantly differ in the frequency or duration of migraine attacks, patients with the ТТ genotype had a higher occurrence of accompanying symptoms (Figure 1), although significant differences were obtained only for photophobia. Besides, patients with the ТТ genotype were significantly (100% of patients) more sensitive to migraine attack triggers (weather changes, irregular sleep, certain foods), as compared to СС genotype (63.6%, p=0.002) and СТ genotype (82.6%, р=0.04) patients.

We also performed a comparative analysis of patient subsets with various *MTHFR* genotypes among MO patients only (64 individuals); this analysis allowed us to demonstrate a significantly higher occurrence of refractory migraine among T-allele carriers (40.6%, 13 subjects), as compared to СС genotype patients (12.5%, 4 individuals), р=0.032.

The average age of homozygous (ТТ genotype) patients undergoing the electrophysiological test (54.2 ± 7.9 years) was significantly higher, than that of the mutation-free subjects (СС 44.3 ± 10.1 р<0.02) or the heterozygotes (СТ 41.8 ± 11.4, р<0.04).

**Figure 2:**
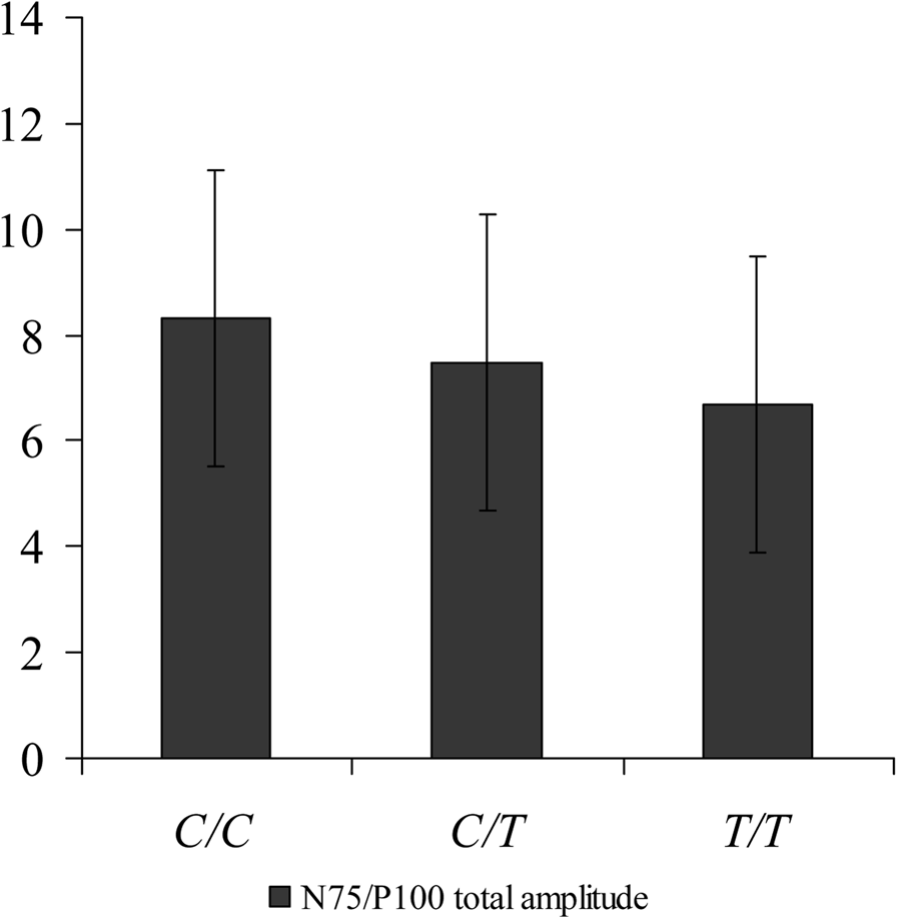
Mean RP-VEP/N75-P100 total amplitude values in the 3 genotype subgroups.

The N75-P100 total amplitude in the ТТ group (6.7 ± 2.8 mcV) was significantly lower, as compared to the СС (8.3 ± 3.6 mcV, р < 0.001) and the СТ (7.5 ± 2.7 mcV, р < 0.01) groups. The N75-P100 total amplitude values in the CT group (mean, 7.5 ± 2.7 mcV) was significantly lower, than that in the mutation-free СС-genotype patients (8.3 ± 3.6 mcV, р < 0.02) (Figure 2).

**Figure 3:**
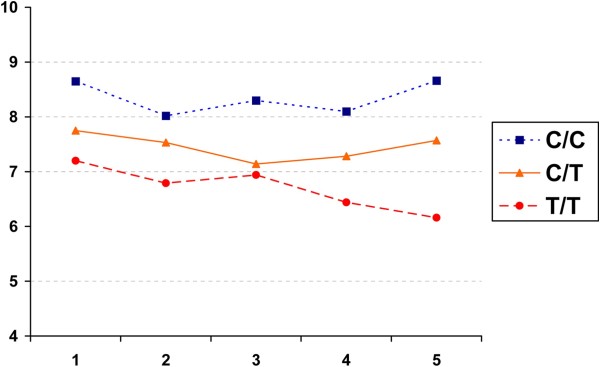
RP-VEP N75/P100 amplitude changes in the 3 genotype subgroups.

Analyzing the amplitude values of the five consecutive stimulus series, we observed the trend of decreasing N75-P100 amplitudes in the Т/Т group in all tests; the difference with the CC group (8.06 ± 1.3 and 8.66 ± 1.56, respectively) attained statistical significance in test blocks 4 (6.44 ± 1.3; p = 0.01) and 5 (6.16 ± 1.1; p = 0.008) (Figure 3, Table 3). No differences were seen in the N75/P100 amplitude between the СТ and the СС groups. However, there was observed a trend to a decrease of N75/P100 amplitude in the СТ group, as compared to the mutation-free patients (СС). Therefore, the patients suffering from migraine without aura and carrying the Т-allele (СТ and ТТ) presented with decreased RP-VEP N75/P100 amplitude levels in all test blocks, which were particularly apparent in the ТТ group.

**Table 3:** RP-VEP N75/P100 amplitude changes and habituation index values in the 3 genotype subgroups

	**Block 1**	**Block 2**	**Block 3**	**Block 4**	**Block 5**	**Habituation index**
**N75/P100 amplitude (μV)**						
**C/C**	8.65±1.60	8.02±1.60	8.23±1.61	8.06±1.30	8.66±1.56	- 0.12%
**C/T**	7.75±0.67	7.53±0.58	7.14±0.59	7.28±0.53	7.56±0.70	+ 2.50%
**T/T**	7.21±1.30	6.79±1.20	6.94±1.60	6.44±1.30	6.16±1.10	+ 17.10%

Analyzing the habituation pattern, we observed a potentiation pattern (relatively stable N75/P100 amplitudes with an insignificant increment in the fifth stimulating series) of the N75/P100 amplitude in patients without the mutation (СС genotype) (− 0.12%). Patients positive for the Т-allele (СТ and ТТ) were found to have a backward trend, which attained the highest level in the ТТ group (+ 17.1%). Significant differences were demonstrated in the habituation index value between the СС and the ТТ groups (p = 0.02).

## Discussion

A relationship between *MTHFR* gene polymorphism and migraine with aura was demonstrated in two completed meta-analyses [2,3]. Similarly, this study demonstrated a significant reliable association between *MTHFR* gene polymorphism and migraine with aura. A detailed analysis of the migraine clinical presentation by *MTHFR* genotype was carried out by Liu A. et al. [10]. They showed that the ТТ genotype was associated with migraine with aura and unilateral headache, the СТ genotype was associated with physical activity discomfort and stress as a migraine trigger. It was also demonstrated that the effect of *MTHFR* gene polymorphism on the clinical picture of migraine was different between males and females. In particular, male patients with the ТТ genotype developed bilateral headache more frequently, as compared to females, and these patients used natural remedies for migraine relief less frequently, whereas female patients with the СТ genotype were more prone to develop migraine accompanying symptoms, such as nausea and osmophobia [10]. In this study, patients with the ТТ genotype had higher rates of accompanying symptoms, regardless of the gender, although significant differences were obtained only for photophobia. Furthermore, this study provided an evidence of the fact that patients with the ТТ or СТ genotypes were significantly more to have sensitivity to migraine attack triggers, as compared to CC patients, which basically goes in agreement with the results of the study conducted by Liu A. et al. [10]. The comparative analysis carried out in this study for groups with various *MTHFR* genotypes among MO patients only (64 individuals) allowed us to demonstrate a significantly higher occurrence of refractory migraine among T-allele carriers.

The MTHFR enzyme catalyzes the transformation of 5,10-methylenetetrahydrofolate into 5-methyltetrahydrofolate, one of the substrates for the homocysteine to methionine transformation. A defect of the thermolabile MTHFR form is accompanied by a moderate hyperhomocysteinaemia. Since homocysteine derivatives act as NMDA receptor agonists, they enhance glutamatergic neurotransmission. Studies conducted both in vitro and in vivo [11,12] have demonstrated that moderate hyperhomocysteinaemia produces a neurotoxic effect. Hyperhomocysteinaemia can predispose cortical neurons in the brain to hyperexcitability. It was shown that *MTHFR* Т-allele carriers with a history of alcohol abuse are at increased risk of generalized withdrawal seizures [13]. This hypothesis was further confirmed by the results of electrophysiological and neurovisualizing studies performed in patients with the *MTHFR* ТТ genotype. In particular, the patients with the ТТ genotype had a significantly lower habituation of the contingent negative variation (CNV), as compared to individuals with the СТ and СС genotypes [8]. The presence of a migraine aura did not affect the CNV habituation level in patients with the *MTHFR* TT variant. At the same time, no differences were seen in the CNV amplitude level between different genotypes in the migraine group. Therefore, the obtained electrophysiological pattern was most likely due to the homocysteine-induced hyperexcitability.

According to the results of previous studies, alterations in *MTHFR* gene (C677T substitution) may lead to an increase in plasma homocysteine levels due to the reduction of enzyme activity. In the recent years, the role of hyperhomocysteinemia in pathogenesis of metabolic diseases and risks of cardiovascular pathology, neurodegenerative disorders, and migraine has been discussed extensively [14]. Several possible mechanisms of hyperhomocysteinemia action may be highlighted: damage of endothelial cells, malfunction in blood coagulation system, direct neurotoxic effects, induction of trigeminal and cortical excitability, and effects on neurotransmitter systems (serotoninergic, noradrenergic).

Few data available on the impact made by *MTHFR* gene polymorphism on neurophysiological parameters are rather contradictory. On the one hand, De Tommaso et al. note a decrease of CNV habituation in TT homozygotes [8]. Authors discuss the role of hyperhomocysteinemia in establishing neuronal hyperexcitability and subsequent CNV habituation deficiency in migraine. On the other hand, Magis et al. showed an absence of changes in VEP habituation in TT homozygotes, as compared to control, and an increase of habituation, as compared to C/T and C/C subgroups [15]. The acquired results may be explained by the activating effects of homocysteine metabolites (DL-homocysteic acid) on serotoninergic transmission.

Our evaluation of the effects of *MTHFR* C677T gene polymorphism on RP-VEP results revealed that the N75/P100 total amplitude was decreased, with statistical significance, in patients with the TT genotype (as compared to the СС and СТ genotypes), and the N75/P100 total amplitude reduction in the CT group was significantly more pronounced than in the mutation-free (СС) subjects. It should be mentioned that the patients suffering from migraine without aura and carrying the Т-allele (the СТ and ТТ groups) were presented with decreased RP-VEP N75/P100 amplitude levels in all 5 stimulation blocks, as well as with positive habituation index values, which were particularly apparent in the patients of the ТТ group. A significant positive impact of the T-allele on RP-VEP test results was demonstrated, i. e. decreased N75/P100 amplitudes and a positive habituation index. These results agree with literature data [15]. The authors present equivalent changes in RP-VEP test results in the patients with C677T *MTHFR* polymorphism. As for the analysis of the numerous neurophysiological data obtained in recent years, one has to take into consideration the fact that these changes in RP-VEP test results (decreased N75/P100 amplitudes and a positive habituation index) are not characteristic of the overall population of migraine patients in their inter-attack period.

It was found that hyperhomocysteinemia (Hcy) leads to impaired lipid peroxidation due to an increase of reactive oxygen species amount and a depletion of antioxidant defense. Moreover, DL-homocysteic acid is an analogue of glutamate and possesses NMDA receptor affinity, which also potentiates the neurotoxic effect of Hcy [16]. In an experimental study, a reduction of amplitude of somatosensory evoked potentials caused by DL-homocysteic acid was also shown [17]. The mechanisms listed above may constitute the basis of a significant decrease of N75/P100 amplitude in the TT homozygotes subgroup demonstrated in our study.

According to Magis et al. [15], a significant reduction in the N75/P100 amplitude and normalization of habituation processes in T-allele carrying patients with migraine may be due to the neurotoxic effect of homocysteine and its derivatives exerted predominantly on serotoninergic neurotransmission.

On the one hand, normal VEP habituation in TT genotype patients can be explained by the hypothesis of a compensatory increase of serotoninergic neurotransmission under the effect of DL-homocysteic acid. On the other hand, similar neurophysiological pattern (a decrease in response amplitudes and normalization of habituation) is typical for ictal period, and it was demonstrated in patients with chronic migraine and medication-overuse headache (МОН) [18,19]. Taking into account that in our study, the frequency of attacks, associated symptoms, and therapy-resistant forms is higher in C677TT homozygotes, we suggest that the decrease of N75/P100 amplitude and normalization of habituation are the reflections of disease chronification. At that, a reduction of dishabituation in TT genotype patients can be caused by neuronal energy deficiency caused by protractedly upregulated serotoninergic transmission and migraine chronification, and by Hcy neurotoxic effects as well. This, in turn, can activate a trigeminovascular system and induce migraine attack.

Although this hypothesis is the most substantiated one at the moment, it still requires further investigation. For future studies, it is important to analyze effects of *MTHFR* gene polymorphism on neurophysiological parameters as a function of dynamics of homocysteine and its general metabolites under therapeutic correction (folic acid/B2/B12).

### Limitations of the study

In the present study, an analysis of plasma levels of homocysteine and its metabolites plasma levels was not performed, and their impact on the neurophysiological parameters was not evaluated. We also did not analyze the neurophysiological parameters of VEP in dynamics as a function of levels of homocysteine and its metabolites plasma levels in plasma under therapeutic correction (folic acid/B2/B12).

## Conclusions

The comparative analysis carried out in this study for groups with various *MTHFR* genotypes among MO patients only allowed us to demonstrate a significantly higher occurrence of refractory migraine in T-allele carriers.

The obtained results showed that patients with TT genotype were not only more likely to have accompanying symptoms (significant differences were found only for photophobia). Moreover, in RP-VEP test results we observed a trend that T-allele carriers were presented with decreased N75/P100 amplitudes and a positive habituation index, as compared to СС genotype patients. Thus, according to our data, the *MTHFR* polymorphism is associated with several clinical and electrophysiological characteristics of migraine.

## Competing interests

The authors declare that they have no competing interests. The authors disclose that they haven’t been influenced by their personal or financial relationship with other people or organizations concerning interpretation of data or presentation of information.

## Authors’ contributions

Research project, Conception: AJE, SАV, KEА, TGR, Organization: AJE, KEА, Execution: AJE, SАV, KLА, SGО, KNS, KZG, Clinical support: AJE, SАV, SKV, FNM, Material support: AJE, SАV, Manuscript Preparation: AJE, SAV, KEA, KLA, Writing of the first draft: AJE, SАV, Review and Critique: KEА, TGR, All authors read and approved the final manuscript.

## Pre-publication history

The pre-publication history for this paper can be accessed here:

http://www.biomedcentral.com/1471-2377/13/103/prepub
